# How to Support Parent-Educators Homeschooling by Open Projects? A Case Study of Two Families Developing Strategic Knowledge

**DOI:** 10.1177/10567879241238361

**Published:** 2024-04-12

**Authors:** Émilie Tremblay-Wragg, Sylvie Viola, Cynthia Vincent

**Affiliations:** 1 Département de Didactique, 14845 Université du Québec à Montréal, Montreal, QC, Canada

**Keywords:** Homeschooling, open projects, strategic knowledge, pedagogical support, COVID-19 pandemic

## Abstract

The COVID-19 pandemic allowed several parents to immerse themselves into homeschooling. In the province of Quebec (Canada), homeschooling is on the rise, increasing from 6,000 children in 2019 to 14,000 in 2021. While integrating learning objectives prescribed by current educational curriculum, parents who act as educators also wish to prioritize pedagogical strategies that are congruent with their preferences and values, such as undertaking open projects involving the whole family. However, these parents face challenges in linking learning goals with their projects. As a result, they require support with project design, among other things, through effective planning, autoregulation, and evaluation mechanisms. Therefore, a study involved six novice families who started homeschooling because of the pandemic impact on the provincial school system. Interviews were conducted with the parent-educators before and after providing them with pedagogical support over a 12-week period, during which a metacognitive guide served as a modelling tool. Our findings reveal that continued support is crucial, particularly for two mother-educators who developed strategic knowledge that set them apart from the other participants. This exploratory study motivates further research on homeschooling, given the renewed interest in this form of education and the need to ensure quality education for all children.

## Introduction

In March 2020, the COVID-19 pandemic caused the closure of 1,900 primary schools in the province of Quebec (Canada), thus forcing close to 900,000 students to overhastily leave school (Ministère de l'Éducation et de l'Enseignement supérieur, 2019). This reality was not exclusive to Quebec, since the pandemic affected more than 1,5 billion students worldwide (Fontenelle-Tereshchuk, 2021; Pozas et al., 2021; Reimers & Schleicher, 2020). Several of these students are now homeschooled in accordance with the Quebec Public Education Act. This unprecedented situation became an opportunity for families to assess if this form of education corresponded to their values and, if so, make the leap toward homeschooling as of the year 2020–2021. When considering the number of children registered for homeschooling in Quebec, during the school year 2020–2021, there is a significant increase (6,000 children in 2019–2020 and 14,000 in 2020–2021). Homeschooling is an education alternative that seemed like an obvious solution, for some parents, to sanitary restrictions imposed during the COVID-19 pandemic. Instead of continuing their schooling in an educational institution, several children started to be educated home with the assistance of their parents adopting the role of parent-educators (Dumond, 2019). By becoming directly involved in their children's education, parents must follow the guidelines established by the Ministry of Education in their educational plans to help children gain knowledge in meaningful and diverse contexts. To that extent, families who have chosen this educational approach, since the beginning of the pandemic, have faced significant challenges, including new considerations for planning learning activities and intervening with their children.

### Homeschooling Projects

According to Pozas et al. (2021), homeschooling allows families, who experiment with this approach, to differentiate their children's learning, enhance their motivation, and appreciate time spent together. A common approach in homeschooling is to engage in projects with the entire family. Projects are frequently utilized in homeschooling because they cater to the children's interests and make it easier to organize the family's schedule around other activities, such as cultural outings and meetings with relatives (Pardo, 2009). Centered on the child, projects foster knowledge building, as well as the capacity and confidence to tackle real-world problems (Scott, 2015). According to several studies, for children to become accomplished citizens, it is essential that they develop useful and enduring competencies (Gauthier & Florin, 2016), which is what projects enable (Scott, 2015). This learning method is not well documented in the scientific literature pertaining to homeschooling. However, the undertaking of projects has been the focus of numerous studies in a formal educational context and demonstrated several benefits (Chen & Yang, 2019). Some of these benefits may include the ability to allocate more than two hours per week to the project (Larmer et al., 2017) or the use of various digital tools (Eskrootchi & Oskrochi, 2010). In this regard, the meta-analysis by Kokotsaki et al. (2016) confirms the advantages of project-based learning in enhancing communication skills, problem-solving abilities, and collaboration. Also, given that the projects undertaken during homeschooling appear to be less formal, time-bound, or subject to strict schedules, it can be assumed that they provide families with more flexibility.

Nevertheless, since 2019, families have faced increased scrutiny from the Department of Education, which requires parent-educators to integrate the disciplinary objectives prescribed by the school program in their teaching. This constraint is seen as disruptive to some families’ *modus operandi*, which prioritizes project-based learning as their educational approach. This situation is concerning because parent-educators who wish to homeschool by projects may not have a thorough understanding of departmental guidelines for the development of critical knowledge and competencies in children. Moreover, they need to learn how to contextualize knowledge and competencies, so that they can be integrated into meaningful projects. As a consequence, parent-educators have expressed the need for pedagogical support to better understand and incorporate these knowledge and competencies into the planning and implementation of their projects.

Between 2020 and 2021, a partnership was established with the *Association québécoise d’éducation à domicile* (AQED)^1^ to conduct a study aimed at supporting six families in the completion of projects that align with departmental critical knowledge requirements. This partnership also sought to increase awareness (strategic knowledge) of parent-educators about their metacognitive strategies for planning, supervising, and evaluating their homeschooling approach. Considering the importance of fostering the autonomy of parent-educators who are solely responsible for their children's education, it was essential to document the development of strategic knowledge for novice parent-educators. In this article, we respond to the following research question: *How does supporting novice parent-educators enhanced the development of strategic knowledge while they complete homeschooling projects with their primary school-aged children?*

## Theoretical Framework

**Figure 1.**
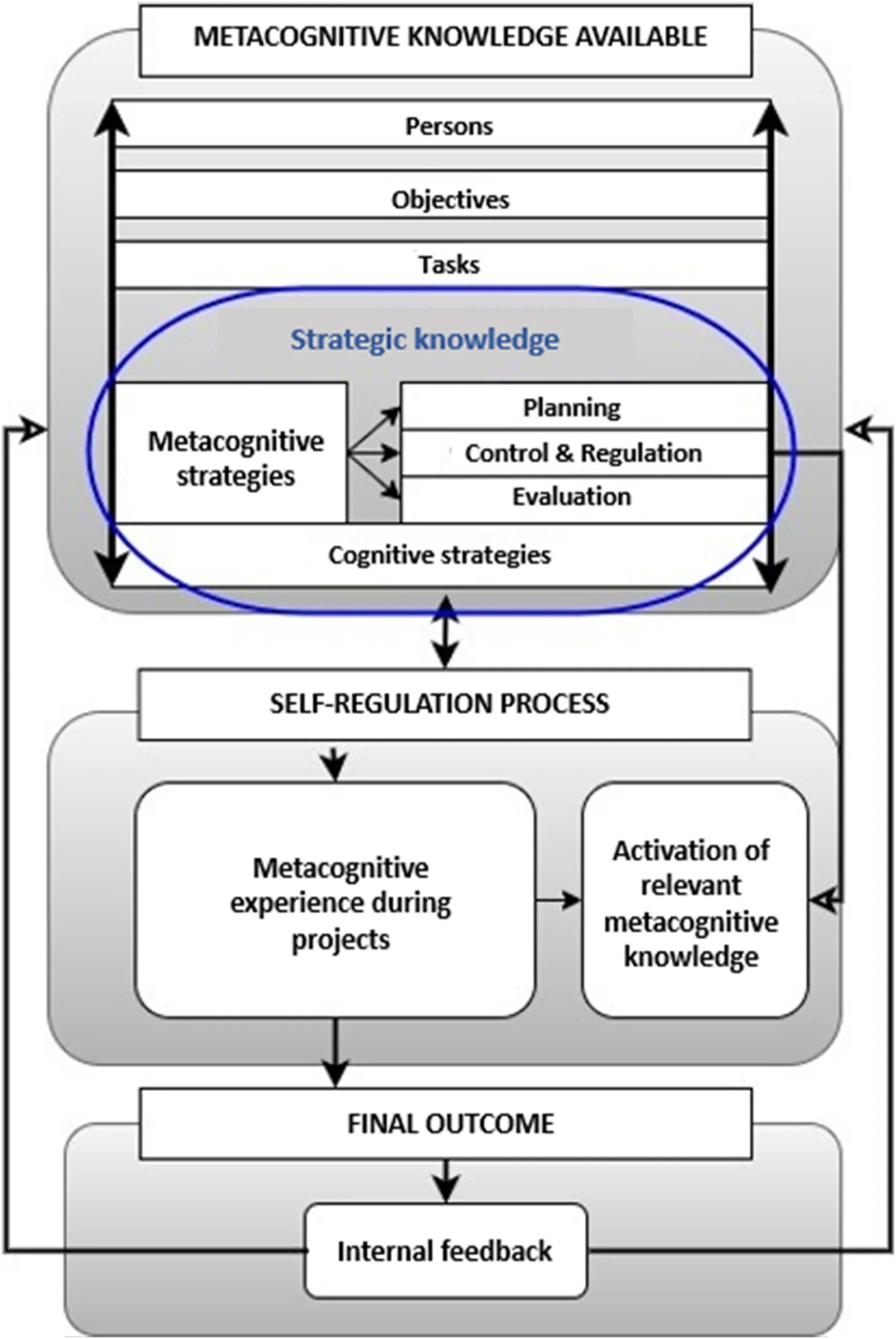
Metacognitive approach to project-based learning, adapted from the framework developed by Lefebvre-Pinard and Pinard (1985).

The undertaking of learning projects provides multiple opportunities for parent-educators to develop their knowledge related to planning and supervising projects with their children. It also affords them the chance to evaluate their own approach as parent-educators. This knowledge is important as it enables parents to gain autonomy and reflect on their practices, among other things, while bolstering their self-efficacy as parent-educators (Ice & Hoover-Dempsey, 2011). This type of knowledge constitutes a critical component of the metacognition concept (Flavell, 1979). Metacognitive knowledge encompasses theoretical knowledge that must be mobilized in practice to validate its relevance for learners. It is also considered to be communicable and can be developed incrementally through experience (Lefebvre-Pinard & Pinard, 1985). Theoretical frameworks on metacognition, including the one proposed by Lefebvre-Pinard and Pinard (1985), stem from studies by Flavell (1979) and Brown (1980), and have been further adapted by scholars from various fields of research, such as Hacker (2018) and Drigas and Mitsea (2021). In this article, the term “learner” refers to a parent-educator, and, as depicted in Figure 1, metacognitive knowledge is characterized by four variables: persons, objectives, tasks, and strategies.

The first variable (*Persons*) refers to what parent-educators know about themselves and others, for example, their interests in a project or their recognition of a pedagogical method that is incompatible with their values. Parent-educators may also understand that planning an engaging activity does not always lead to desired outcomes if the level of difficulty is too high or the content is not suited for their children. The second metacognitive knowledge variable (*Objectives*) involves parent-educators formulating clear and realistic goals in relation to the projects they present to their children. The third variable (*Tasks*) corresponds to the parent-educators’ knowledge of the task at hand, including essential subject areas for their children's education or which tasks are more demanding. The fourth and last variable (*Strategies*) encompasses cognitive and metacognitive strategies, also known as “strategic knowledge.” Cognitive strategies help individuals to solve problems, whereas metacognitive strategies enable them to monitor progress while completing a task. The latter allow the planning, control, regulation, and evaluation of cognitive strategies deployed to reach an established goal (Lefebvre-Pinard & Pinard, 1985). The activation of metacognitive knowledge occurs during a metacognitive experience when a person adjusts their knowledge repertoire. The metacognitive knowledge is then enhanced, better equipping the individual to handle new experiences. In our study, we concentrate on the development of the parents’ strategic knowledge when they plan, intervene, and reflect on their own practices while designing and executing educational projects with their children. This focus is rationalized by the fact that we can readily query and observe the mobilization of strategic knowledge in real-time, compared to other types of variables.

## Methodology

This section presents the participating families and research type, support mechanism, data collection and instruments, as well as our data analysis method.

### The Participants and Type of Research

For the purpose of this research, six novice families were selected through a three-step process. First, all families with AQED membership were solicited through a private Facebook group and through the organization's newsletter via email. Then, interested families were invited to fill out an online questionnaire that included eligibility questions. These families would have started homeschooling during the school year 2020–2021 due to the COVID-19 pandemic. Moreover, they had to demonstrate a motivation to receive pedagogical support from a research team while completing projects on the website ecollaboration.uqam.ca. A total of seven families responded to the questionnaire, but one withdrew prior to the final selection step. According to Fortin and Gagnon (2022), only one unit of analysis is sufficient to conduct a case study for the purpose of developing an in-depth understanding of the studied phenomenon. In this article, we analyzed the experience of two families that set themselves apart from the sample due to their perseverance in completing open projects and their diligence in taking measurements. These two families completed three projects (60 min of pedagogical support on average per week for each family) while the other four families finished between zero and two projects (30 min of pedagogical support on average per week for each family). The case study was chosen as the explorative research type since the research objective (metacognition in a homeschooling context) is a complex and restricted phenomenon, and the focus was on its description (Merriam, 1988). Table 1 presents the profiles of the two novice families.

**Table 1.** Profiles of Two Novice Families Homeschooling Their Children (Created by Authors).

First names of parent-educators	Number of school-aged children	First names and ages of children	Living environment	Level of education of the parent-educator	Number of completed projects *Écollaboration*
Chloe	3	Zara: 6 years old Matteo: 7 years old Mary: 9 years old	Rural	Bachelor in Business administration	Chocolate Warm Up Miniature Garden Comical Transformation
Florence	2	Angy: 7 years old Debora: 8 years old	Rural	College diploma in Arts and Letters + Undergraduate microprogram in glass work	Edible Masterpiece Living Board Animal Simulation

The two selected parent-educators reside in Quebec and have three and two primary school-aged children, respectively. It is worth nothing that the mothers assumed the main role in participating in the different phases of the study, which is not uncommon in homeschooling as a high proportion of women take on the responsibility of educating children (Slater et al., 2022). Therefore, in the Results section of this article, the term “mother-educator” will be used to refer to the participants.

### The Support Mechanism

**Figure 2.**
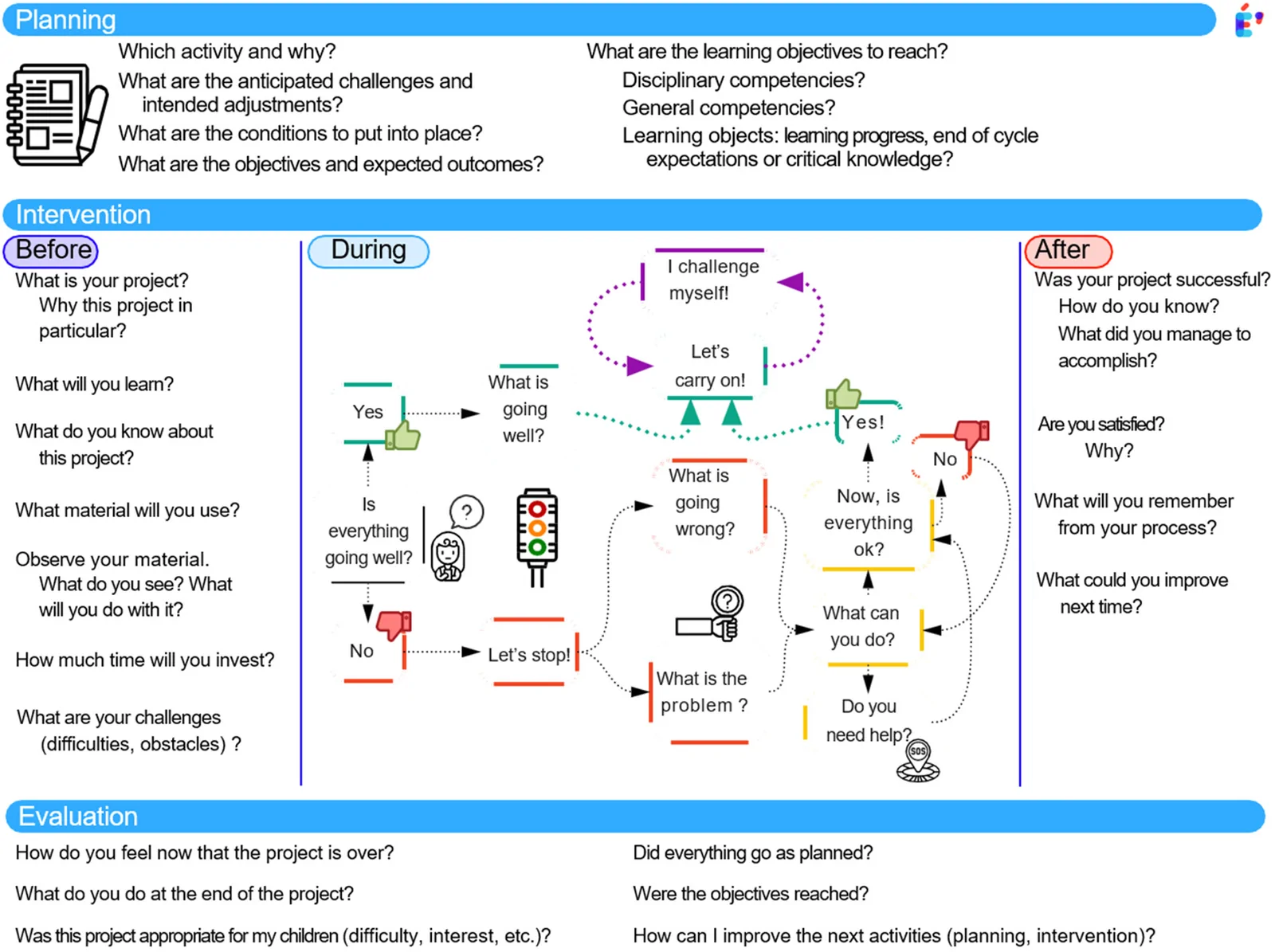
Metacognitive guide for parent-educators adopting a project-based learning approach to homeschooling.

The support mechanism in the center of this study was developed to assist novice parent-educators design and execute projects that align with learning objectives required by the Department of Education. To that end, weekly virtual meetings lasting one hour were organized for a 12-week period using the Zoom platform. The meetings were led by two third-year undergraduate students from the Bachelor program in Education at preschool and primary schools at Université du Québec à Montréal. These students guided parent-educators through a process of reflection on their homeschooling practices, including planning, intervening with their children, and evaluating their own approach. The research team also developed a metacognitive guide (Figure 2), which was explained to the parent-educators at the start of the study. This instrument benefited not only the facilitators, but also the parent-educators throughout their participation.

#### The Metacognitive Guide

This guide favors an approach that encourages parent-educators to develop a reflexive posture toward the three key phases of a teaching program (Planning, Intervention, and Evaluation).

The first phase related to *Planning* includes all steps required for parent-educators to design a learning project. Questions are identified to help self-reflection (i.e., *What are the learning objectives that this project aims at?*). During this phase, everyone's interest and anticipated challenges are also considered. The second phase, that is *Intervention*, finds itself at the heart of the metacognitive guide. Parent-educators participate in the development of learning strategies by posing clear and relevant questions to their children according to three project steps (before, during, and after). For example, during the project, parent-educators could ask the question: *Is everything going well?* if they wanted their children to become aware of their difficulty or ease to complete a task. Then, they can follow the sequence of questioning depending on the children's responses. The third and last phase, which is the *Evaluation,* revolves around the parent-educators’ appreciation for their own planning and intervention. For instance, they can ask general questions about project planning, as well as their support provided to children during the intervention phase.

#### The Écollaboration^2^ Projects

The projects used in our study were learning activities designed by our research team and placed on the website: ecollaboration.uqam.ca. To develop these activities, we considered the definition of project-based learning, which suggests that learners build their own knowledge by sharing the results of their research with their peers while working on a final product to achieve a common goal (Bell, 2010). In total, 26 projects were designed on various themes that were deemed to be both engaging and universal (i.e., nutrition). Our team also aimed to create projects that corresponded to authentic family situations and, at the same time, allowed learners to develop general competencies (Scott, 2015). Over the course of 12 weeks, families received pedagogical support, and had to complete three projects of their choice from 26 available options, which are listed in Table 1 with their hyperlinks.

### The Instrument and Data Collection

In order to document the development of parent-educators’ strategic knowledge, we conducted semi-structured interviews with both families before and after they received pedagogical support. The majority of the questions related to the strategic knowledge of parent-educators according to the three phases of a project. For example, we were asking the following questions regarding the planning phase: *What do you do when you prepare a project for your children?* When addressing the control and regulation phase, one of the questions posed was: *What do you do when a project does not progress as you anticipated?* As for the evaluation phase, this question was used: *What do you do when a project is completed?* The results presented below highlight the difference in perception of mother-educators by comparing their initial and final interviews.

### The Method for Data Analysis

The analysis of qualitative data collected was completed by using a mixed coding matrix, that is a list composed of three themes (1-planning, 2-intervention, 3-evaluation) and 15 sub-themes from the theoretical framework (metacognitive knowledge variables). Other themes (98) directly emerged from both families’ interviews. We coded all pertinent excerpts by using the aforementioned matrix, in line with the manifest content analysis method proposed by Van der Maren (2003). The third author of this article was responsible for conducting all interviews and coding data, which ensured uniformity in the way data were analyzed.

## Results

In order to present the evolution of Chloe and Florence cases, findings from this explorative study are divided into two sections, namely the metacognitive strategies reported prior to participants joining the Écollaboration research project, then strategies of the same nature reported and witnessed after the 12-week pedagogical support. These sections highlight three types of metacognitive strategies: planning; controlling and regulating; evaluating, which are identified in bold fonts in the text below. Moreover, the four strategic knowledge variables are depicted in a table summarizing their relation to each of the two mother-educators.

### The Strategic Knowledge of Mother-Educators Prior to Receiving Pedagogical Support

During the first interview, Chloe self-assessed as a novice in regard to *planning*, a competency that she wanted to develop by participating in the research. More specifically, she doubted the efficiency of her homeschooling program: “It is just the uncertainty about knowing if what I do is really good, but so far, it works. It should not be too bad (laugh).” At the start of the project, Chloe mentioned seven strategies that she appeared to be using. Among others, she conducted searches to find resources and ideas about learning activities that “could be suitable for all three [children] at once.” To do so, she surveyed the internet, departmental documents, and youth scientific journals to which she subscribed. Additionally, she relied on her experience to teach some school subjects, such as reflected in the following excerpt:

I used to work as a baker. So, fractions in baking, they are very useful. So, we baked muffins. They did not think that it was a project, but while they made the muffins [they learned] how to calculate, how to double recipes. We practised multiplications and fractions with that. It was *incognito*.

Similarly to Chloe, when Florence planned, she started by “a little bit of research” on learning activities related to her children's interests. The children were involved in the research phase prior to starting activities: “For example, if [my girls] want to bake a cheesecake, we will search for a recipe together. Then, we will figure out the list of required material.” Moreover, she looked for ways to broach current issues and personal values, such as healthy habits, eco-responsibilities, openness to the world, etc.

In terms of *control and regulation*, Chloe explained that when her children undertook projects, she usually started with providing instructions on the task to complete. Considering that autonomy is an important value, she encouraged her children to become independent, for example, by not getting involved when they were problem-solving. In this respect, Chloe recognized when something went wrong. In those instances, she mobilized strategies to assist her children, while reflecting on her interventions in real-time: “When it is time for recess, I revise what I have done. If I think that it made sense, or that the children are too hyper, then, we meditate.”

Florence, on the other hand, supervised her children too much, which is something that she wanted to adjust, as attested in this excerpt: “Yesterday, Deborah was completing her little project on colors and words, and I was sitting next to her, when I realized that I could be in the kitchen and exchange with her from a distance.” She regularly reassured her children and asked questions, instead of offering avenues to explore solutions. Florence also mentioned that she gauged the interest of her children throughout the projects. She considered projects to be successful if she noticed that her daughters were laughing and exchanging with each other.

During her initial interview, Chloe explained her strategies to *evaluate* her teaching program and her interventions. She provided a rather nuanced response to the question: *How do you feel when you complete a project with your children?* When a homeschooling project do not provide the expected outcomes, she recognizes that it is exhausting, but she realizes that it is an opportunity to learn from her mistakes. Furthermore, to evaluate if the project is successful or not, she reports simply observing her children's reaction: “When you propose an experience to them and their jaws drop, we understand that we did things wrong.” In short, when everything goes well, it reinforces her choices to homeschool. When asking Florence the same question, she answered that she was uncertain about when her children reaches the learning goal. However, she was already aware that the notion of a successful project is relative.

### The Metacognitive Strategies of Mother-Educators After Receiving Pedagogical Support

After receiving pedagogical support, Chloe explained that her *planning* was more systematic by programming hourly blocks, which was a different way of operating than the one previously used. According to her account, it is thanks to the support received that she was able to understand the variety of knowledge to introduce in projects. This is also a notion that was “nebulous” to her beforehand since she was “used to a traditional approach.” The mother-educator now plans by integrating subjects more organically, for example, she introduced decimals in the *Chocolate Warm Up* project. In doing so, her children were able to calculate their shopping budget. Furthermore, the selection of a project now takes into account its duration, as well as the children's interests and attention span, as per stipulated in the following excerpt:

It starts with the selection. I look at those that are not too long. [One of my children], if it drags on, I will lose him. There is nothing I can do about it. We found out that he has an attention deficit disorder, and we were told that quick rewards are more beneficial to him than something that drags on.

In the same vein, Florence mentioned realizing that planning starts by identifying her children's interest. She spent time reviewing the description of projects with her daughters to inform them of the selected project scope and required material. She also specified learning objectives. Additionally, Florence noticed that she should avoid steering her choices of projects according to disciplines. This new way of operating results in projects that are more “creative and artistic” than what she could have imagined before participating in our study. The children's interest level is the factor that still matters most, but she also allows herself to challenge them, to “push them further.”

During her final interview, Chloe shared her *control and self-regulation* strategies that she learned and exercised throughout her participation in our study. In that respect, she mentioned asking her children questions to further their reflection or bring their focus back on the task at hand when they were distracted. Otherwise, she attested developing her capacity to better align her intervention to her children's ages, thus allowing more “autonomy and laisser-faire,” as per mentioned in this excerpt: “At first, I would say that it was reassuring to maintain control. […] When we went to the flower shop to do the miniature garden, I did not intervene at all. They sorted out everything with the florist.”

When the activity started to go off track, she intervened to bring the focus back on the required task: “Group, let's focus!” Furthermore, she used positive reinforcement and metaphors to explain what great inventors experienced when they faced challenging situations. This approach promoted patience and perseverance: “I showed her how many times the lightbulb inventor failed, how many times it took him to succeed. Even with big brains, it is difficult.” Lastly, she invited other family members to get involved to bridge her own shortfalls while completing projects. For example, she asked her spouse to look after the miniature garden project, because she does not have a green thumb.

Florence also expressed her willingness to provide her children with greater autonomy. She articulated this new approach in the form of a goal:

In the “during,” I try [to intervene] as little as possible. […] I tried to move away while [my daughters] were creating. I believe that there has been an evolution in my involvement, but they often ask for [my help].

Additionally, when Florence noticed problems in the way activities were progressing, she assisted her daughters by asking them questions to reflect on the situation. Florence also explained that she and her daughters conducted internet searches on how to complete projects. In more difficult cases, she helped and guided her children by first demonstrating the different steps to follow before letting them complete these same steps on their own. She offered continued support and intervened, on occasion, to assist her daughters to reach their goals.

In regard to *evaluation*, Chloe mentioned that she is responsible for engaging her children into projects. Concretely, as a strategy, she now easily succeeds to integrate school notions while working on projects when everyday situations are relevant: “now, it seems more instinctual.” In addition, Chloe noticed that she understands how to pinpoint the interest of a child. She also lets knowledge “simmer” before addressing it again at a later time. Being a homeschooling novice, she realized that it is important to get out of her comfort zone. She believes it is the parent who must adjust to avoid reproducing old practices because “we are really inclined to copy paste what we experienced.” Furthermore, she reaffirmed the importance of letting children work autonomously on projects and to learn to let go: “As a parent, it is difficult to pull back. We want to do things the right way, on their behalf. Not to aim for perfection, but [rather] aim for autonomy.” Florence recognized that she learned a lot as a mother-educator. She described the evolution of her vision when integrating learning objectives in projects. This mother-educator shared her values related to the importance of persevering to complete a project in which her entire family is involved.

Several other strategic knowledge elements resulted from the data analysis. Table 2 illustrates a summary of strategic knowledge development for both mother-educators, prior to and after receiving pedagogical support, by distinctly comparing the three categories of metacognitive strategies.

**Table 2.** Overview of Strategic Knowledge Development for Two Mother-Educators (Created by Authors).

	Prior to receiving pedagogical support	After receiving pedagogical support
**Metacognitive strategies used by Chloe**		
Planning	Doubted her planning process Researched resources and ideas Established links with her experience	Plans set periods of activities Integrate learning goals Selects shorter projects Considers children's level of interest and attention span
Control and regulation	Provided instructions Stood back when problems arose Proposed recess periods Reviewed interventions Suggested to meditate	Asks questions to further her reflection Gets children to focus when they are distracted Adapts her interventions to children's age level Promotes autonomy and independence Offers tailored feedback Employs positive reinforcement Utilizes metaphors for ease of understanding Values patience and perseverance Solicits other resource persons
Evaluation	Recognized her exhaustion Learned from her mistakes Observed her children's reactions Validated her choice of education	Realizes her responsibility to motivate her children Integrates school notions in projects Evaluates her capacity to ascertain her children's interest Lets the activity simmer to get back to it later Realizes the importance of getting out of her comfort zone Accepts to evolve in her teaching Reaffirms the importance of working on her children's autonomy Values perseverance Adopts an approach of letting go
Metacognitive strategies used by Florence		
Planning	Researched resources and ideas Planned materiel Promoted personal values Considered current issues	Considers the children's interest Reviews the description of projects Informs children of the project scope Plans materiel Specifies learning objectives Does not steer projects according to disciplines Proposes challenges
Control and regulation	Supervised her children Reassured her children Asked questions Surveyed interest level Assessed the project execution	Promotes autonomy and laisser-faire Notices her children's difficulties Asks questions to further reflection Involves her children into internet searches for resources Models and supports when a task is more challenging
Evaluation	Did not know how to evaluate reaching goals Understand the successful completion of a project	Realizes her own planning competencies development Values perseverance

Certain strategies used by mother-educators prior to receiving support remain afterwards, whereas new strategies supplement those now available for mobilization. In that respect, Table 2 illustrates the development of mother-educators’ strategic knowledge by showing the increased number of metacognitive strategies recognized after receiving support.

## Discussion

This study aimed at documenting the strategic knowledge development of novice parent-educators who received pedagogical support to undertake homeschooling projects with their children of primary-school ages. Three key findings stand out regarding the evolution of strategic knowledge during the planning, control and regulation, and evaluation phases.

### Parent-Educators Can Learn to Plan Projects that Integrate Learning Objectives From the Current Educational Curriculum, As Well As Their Children's Characteristics

According to Aldabbus (2018), one of the main challenges of project-based learning is to establish links with the educational curriculum. Therefore, our exploratory study reveals innovative results related to novice parent-educators and their ability to integrate learning objectives of the school program when planning homeschooling projects, while taking into account their children's characteristics.

As previously mentioned, it was the first homeschooling experience for both mother-educators that involved planning and exploring project-based learning. Prior to receiving pedagogical support, planning was done in a spontaneous fashion depending on available resources, such as activity manuals that are largely used in homeschooling environment (Hanna, 2012). Even though previous research showed that providing support to families may result in improving their teaching practices, our study revealed that the pedagogical support received makes the main difference. In that respect, novice mother-educators mentioned that it was difficult to consider other approaches than traditional teaching because it was what they knew best, but they realized that this method was not applicable to homeschooling as mentioned before by Ortloff (2006). A literature review of 351 documents conducted by Kunzman and Gaither (2013) suggested that after one or two years of homeschooling experience, mother-educators progress toward a less structured and more eclectic approach, which requires of the parent-educator a capacity to create learning experiences (Aldabbus, 2018).

On another note, after receiving pedagogical support, both mother-educators mentioned that projects are now selected based on the children's interests by offering them a chance to decide what they want to do. It is what was recommended by Purwaningsih and Fauziah (2020) when homeschooling. Moreover, parent-educators are able to organically insert the required learning content into projects compared to their previous planning practices. This allows new pedagogical possibilities to emerge during projects. In this case, parent-educators adjust their established plan and upcoming activities according to their children's characteristics, namely their interests. As a consequence, mother-educators view their planning as an iterative process. Therefore, they are able to evaluate and modify their plan according to the project scope, as well as the children's characteristics and attention span.

In sum, mother-educators expanded their metacognitive strategies associated with project planning after receiving pedagogical support. While they were doubting their planning practices before participating in our study, they now demonstrate increased flexibility with integrating learning objectives from the educational curriculum. According to Carpenter and Gann (2016), this improvement, combined with their enhanced consideration for their children's characteristics, represent an element that mother-educators appreciate when homeschooling.

### Parent-Educators Can Learn to Regulate Their Interventions to Benefit Their Children

As specified, autoregulation consists of an intervention by parent-educators when children face challenges, by asking them questions, so that they identify the nature of the problem and find their own solutions. While pedagogical support was provided, our research team presented and modelized the *Metacognitive guide* (Figure 2) to assist parents with refining their autoregulation strategies. What stands out the most from these new strategies deployed by mother-educators is that questions posed to children foster their reflection and autonomy, which goes hand-in-hand with the capacity of the parent-educator to let go, similarly to what was described by support group participants in a study by Dumond (2016). Thanks to the support received, mother-educators gained confidence in assisting their children to become better thinkers. They are now able to ask relevant questions and assist their children during the completion of projects. Therefore, it is safe to say that they learned to play a role of facilitator, adviser, and guide, as per stipulated by Aldabbus (2018). They were also able to observe more than what they did in the past, as per recommended by Proulx (2004) when teachers undertake a project-based learning venture.

### Parent-Educators Can Learn to Evaluate Their Own Practices

In our study, evaluating means that parent-educators reflect on their capacity to plan and regulate their interventions when completing a homeschooling project. Our findings presented in the above section highlighted an important observation broken down into two analytical perspectives: the evolution of both mother-educators’ perceptions toward their planning capabilities and their realization that they developed new autoregulation strategies.

Our findings first revealed that the two mother-educators noticed an improvement with their project planning competencies. In this regard, Chloe gradually modified her perceptions by adopting a more open and constructive posture, even though she originally regarded her planning abilities in a negative light. She seemed ambivalent between the workload that homeschooling entails and its benefits. This significant workload experienced by mother-educators is reported as a homeschooling disadvantage in the dissertation by Baker (2021). In addition, Chloe mentioned that before receiving pedagogical support, she felt exhausted and that she was operating by trials and errors. After receiving support, she adopted a much more flexible posture and became a reflexive educator, that is regarding “lâcher-prise” and the importance of getting out of her comfort zone to undertake larger projects. In a sense, she allowed herself to evolve as a pedagogue. As for Florence, even though she only addressed this aspect briefly, she also recognized the development of her planning competencies. According to Wall et al. (2015), when parent-educators are enthusiastic and ready to experiment with different projects with the entire family, metacognitive experiences or activities are more frequent and enriching.

The second analytical perspective focuses on tools facilitating metacognitive questioning and the development of planning and autoregulation strategies. Through their participation to our study, mother-educators noticed the development of new strategies, among other things, by using the metacognitive guide, asking questions when assisting children, and using the recommended scaffolding process. This observation aligns with the work of Alston-Abel and Berninger (2018) mentioning that questioning parent-educators encourages them to reflect on and engage with their own pedagogical practices. Moreover, the pedagogical support received allowed them to effectively intervene with their children by using similar reflexive questions pertaining to the process instead of the product, which corresponds to the goals of a project-based pedagogy (Kokotsaki et al., 2016).

### Limitations

It is important to consider the limitations of our study. Firstly, the data collected are based on self-reported accounts by mother-educators interviewed. Secondly, this article presents the experience of two families out of the six families to which we provided pedagogical support. The other four families expressed concerns with the combined workload of homeschooling and participating in our study, as their main justification for withdrawal. Moreover, they mentioned personal and family related reasons for their lack of involvement, such as medical issues, high-risk pregnancy, etc. It is our assessment that the virtual nature of the pedagogical support provided, and the context of the COVID-19 pandemic also impeded the commitment of some families. In this regard, if in-person support had been provided in the natural family environment, we consider that closer connections could have been established between researchers and mother-educators, leading to enhanced perseverance on the part of the families seeing a greater value in the project. In sum, if the overall conditions had been more favorable, we could have spent more time with families to provide tailored support for their usage of the proposed tools to the benefit of all participants. Therefore, it would be ill-advised to generalize the results presented above since the two selected families were unique and exemplary cases within the group of novice families participating in our homeschooling study.

## Conclusion

This article aimed at reporting findings from a study conducted in the context of homeschooling during the COVID-19 pandemic, for the purpose of supporting six families considered novice in the development of parent-educators’ strategic knowledge. The specific strategic knowledge profile of two mother-educators relating to planning, self-regulation, and evaluation during the completion of family projects highlighted the benefits resulting from a 12-week pedagogical support program. In view of the limited studies examining parents’ strategic knowledge in homeschooling environments, our research highlighted the importance of examining this phenomenon, especially with the compounded requirements and interests toward this mode of education due to the recent sanitary crisis. Therefore, we regard this field of research as vital to facilitate the undertaking of open projects by parent-educators, who will confidently integrate learning objectives prescribed by the existing school program to optimize their children's education. To that end, it is essential to increase and tighten the collaboration among stakeholders involved in homeschooling (universities, schoolboard leaders, government administrators, AQED members). A common vision will then be conveyed during training programs for administrators of homeschooling directorates and in parent-educators’ support networks to promote meaningful and contextualized learning.

## Notes

1. Translation from French to English: The Quebec Association for Homeschooling.

2. Écollaboration is a play on words between “école” meaning “school” in French and “collaboration.”
